# Delayed reendothelialization with rapamycin is rescued by the addition of nicorandil in balloon-injured rat carotid arteries

**DOI:** 10.18632/oncotarget.12444

**Published:** 2016-10-04

**Authors:** Ying Qian Zhang, Feng Tian, Jin Song Chen, Yun Dai Chen, Ying Zhou, Bo Li, Qiang Ma, Ying Zhang

**Affiliations:** ^1^ Department of Cardiology, Chinese PLA General Hospital, Beijing, China; ^2^ Department of Cardiology, Chinese PLA 175th Hospital, Fujian, China; ^3^ VIP Medical Service Department, Beijing Shijitan Hospital, Beijing, China

**Keywords:** nicorandil, rapamycin, xanthine oxidase, endothelium, angioplasty

## Abstract

Rapamycin is an immunosuppressive agent that is added to drug eluting stents. It prevents restenosis, but it also impairs reendothelialization. Nicorandil is a hybrid agent with adenosine triphosphated (ATP)-sensitive K^+^ (K_ATP_) channel opener and nitrate properties. It prevents oxidative stress and cell apoptosis induced by rapamycin in endothelial cells *in vitro*. However, whether nicorandil promotes reendothelialization after angioplasty delayed by rapamycin remains to be determined. Balloon injury model was established in SD rats. Nicorandil increased reendothelialization impaired by rapamycin, and it decreased xanthine oxidase (XO)-generated reactive oxygen species (ROS) induced by rapamycin. In addition, eNOS expression inhibited by rapamycin was increased by nicorandil *in vivo*. *In vitro*, rapamycin-impeded cardiac microvascular endothelial cells (CMECs) migration, proliferation and rapamycin-induced ROS production were reversed by nicorandil. Knockdown of XO partially inhibited rapamycin-induced ROS production and cell apoptosis in CMECs, and it promoted CMECs migration and proliferation suppressed by rapamycin. Knockdown of Akt partially prevents eNOS upregulation promoted by nicorandil. The beneficial effect of nicorandil is exhibited by inhibiting XO and up-regulating Akt pathway. Nicorandil combined with rapamycin in effect rescue the deficiencies of rapamycin alone in arterial healing after angioplasty.

## INTRODUCTION

Drug eluting stents (DESs) are developed for preventing restenosis after percutaneous coronary intervention (PCI). However, clinical studies suggest that DESs delay reendothelialization, and DESs appear to be accompanied by a higher prevalence of stent thrombosis [1]. Rapamycin is an immunosuppressive agent that is added to DESs. It inhibits vascular smooth muscle cell proliferation and migration to prevent restenosis [2]. However, it also induces autophagy and apoptosis in endothelial cells [3], increases oxidative stress [4], and thus suppresses healing of the endothelium [3].

Endothelial cells originating from intact adjacent arterial segments populated the stent luminal surface as a result of local proliferation and migration [1]. Nicorandil is a hybrid agent with adenosine triphosphated (ATP)-sensitive K^+^ (K_ATP_) channel opener and nitrate properties [5]. Clinical studies find that nicorandil reduces the rate of target vessel revascularization [6]. Preclinical studies revealed that nicorandil attenuates endothelial VACM-1 expression in diabetic rats [7], prevents rapamycin-induced production of reactive oxygen species (ROS) [8], promotes eNOS expression [9], and inhibits endothelial cells apoptosis [10]. Nicorandil also increases endothelial cells proliferation and migration [11]. Nicorandil may promote early phase of reendothelialization that is delayed by rapamycin.

ROS generated in coronary arteries impairs reendothelialization [12]. Xanthine oxidase (XO) is identified as a source of ROS in cardiac microvascular endothelial cells (CMECs) [13], atherosclerosis [14] and coronary disease [15]. Rapamycin could directly activate XO [16]. Nicorandil has been found to inhibit XO activity in diabetic rats [17]. Thus, nicorandil may promote reendothelialization by inhibiting XO-generated ROS. eNOS is the dominant enzyme that contributes to the production of nitric oxide (NO) in vessels [4], and is an indicator of reendothelialization [18]. Rapamycin inhibits eNOS expression *in vitro* [4], but nicorandil promotes eNOS expression [9]. We build the hypothesis that nicorandil may promote reendothelialization impaired by rapamycin through inhibiting XO-generated ROS formation and promoting eNOS expression. *In vivo* carotid balloon injury model and *in vitro* CMECs were used to indentify this hypothesis.

## RESULTS

### Nicorandil promotes delayed reendothelialization induced by rapamycin

**Figure 1:**
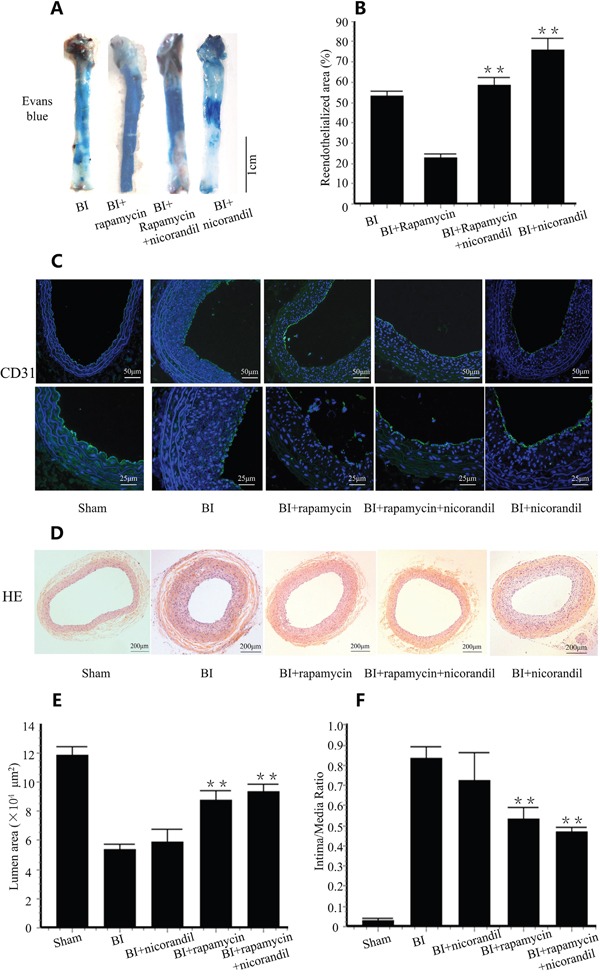
Nicorandil promotes reendothelialization impaired by rapamycin **A.**, SD rats received BI procedure were randomized to vehicles, rapamycin, nicorandil or rapamycin-nicorandil co-treatment. Reendothelialization area is the area that does not uptake Evans blue dye. **B.**, Summary data for reendothelialization is expressed in percentage. Values are mean±SE. **p<0.01 vs. rapamycin group. **C.**, Immunostaining of PECAM-1 (CD31) in rats treated with vehicles, rapamycin, nicorandil or rapamycin-nicorandil co-treatment. **D.**, Cross sections of carotid arteries in different groups 14 days after BI. Sections were stained with H&E. **E.** Quantitative analysis of lumen area. Bars represent means±SE. **p<0.01 vs. BI group. **F.** Quantitative analysis of intima to media area ratio. Bars represent means±SE. **p<0.01 vs. BI group.

Reendothelialization was valued by Evans blue staining and immunostaining of PECAM-1 (CD31). 14 days after balloon injury, 53.1±0.03% of the injured lumen surface restored reendothelialization in balloon injury (BI) group. Rapamycin impaired reendothelialization. Nicorandil itself significantly promoted reendothelialization. Co-treatment of nicorandil promoted reendothelialization impaired by rapamycin from 22.5±1.8% to 58.3±4.0% (p<0.01) (Figure 1A and 1B). PECAM-1(CD31) positive cells in lumen surface showed a partial restoration of the endothelial cell monolayer in BI group, and rapamycin decreased this restoration. In contrast, the PECAM-1(CD31) positive length in lumen surface was accelerated by co-treatment of nicorandil (Figure 1C), consistent with the results of Evans blue staining. The effects of nicorandil and rapamycin on intimal hyperplasia were also evaluated. 14 days after balloon injury, intimal hyperplasia developed in BI group, whereas it was suppressed in rapamycin group (Figure 1D). Lumen area in rapamycin group was larger than that in the BI group (8.82±0.711 vs. 5.34±0.354 × 10^4^μm^2^, p<0.01) (Figure 1E), and intima to media area ratio was significantly smaller than that in the BI group (0.535±0.059 vs. 0.86±0.061, p<0.01) (Figure 1F). Co-treatment of nicorandil and rapamycin showed a larger lumen area and a smaller intima to media area ratio than the rapamycin group, but there were no statistically significance (p>0.05).

### Nicorandil inhibits oxidative stress in carotid arteries

**Figure 2:**
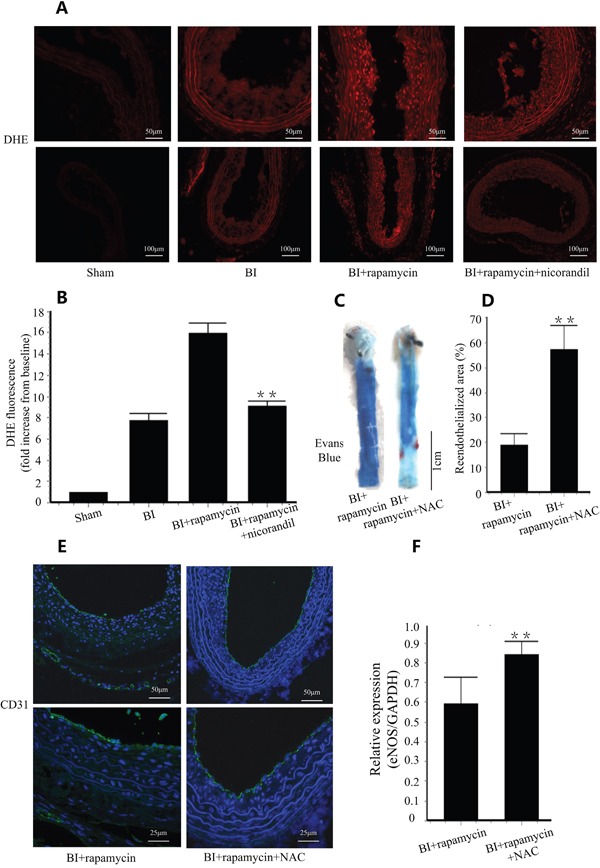
Nicorandil inhibits in situ oxidative stress **A.**, In situ detection of ROS production with DHE staining. **B.**, Summery data of DHE fluorescence in difference groups. Values are mean±SE. **p<0.01 vs. rapamycin group. **C.**, Reendothelialization was valued by Evans blue staining in each group. NAC (150mg/kg/day) was given by gavage feeding. **D.**, Summary data for reendothelialization is expressed in percentage. Values are mean±SE. **p<0.01 vs. rapamycin group. **E.**, Immunostaining of PECAM-1 (CD31) in lumen surface. **F.**, eNOS expression in carotid arteries were analyzed by qRT-PCR. Values are mean±SE. **p<0.01 vs. rapamycin group.

We detected ROS production in carotid arteries by DHE staining. BI group showed a high fluorescence signal in intima area and rapamycin further increased ROS production. However, co-treatment with nicorandil significantly decreased ROS production (p<0.01) (Figure 2A and Figure 2B). To confirm the role of ROS in rapamycin-impaired reendothelialization, NAC, a cell permeable antioxidant was used as additional treatment. NAC accelerated reendothelialization impaired by rapamycin from 19.9±4.45% to 56.7±7.22% (p<0.01) (Figure 2C and Figure 2D). PECAM-1 (CD31) positive length in lumen surface and eNOS expression in carotid arteries were also accelerated by NAC (Figure 2E and Figure 2F), consistent with the results of Evans blue staining.

### Nicorandil inhibits XO production and promotes eNOS expression in carotid arteries

**Figure 3:**
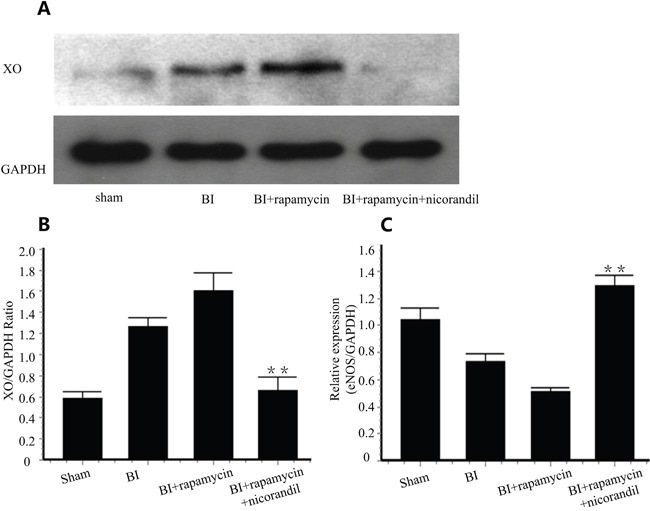
Nicorandil inhibits XO production and promotes eNOS expression **A.**, Representative western blots for XO in injured carotid arteries treated with or without rapamycin or nicorandil. **B.**, Quantitative analysis of XO protein level. Values are mean±SE. **p<0.01 vs. rapamycin group. **C.**, eNOS expression in carotid arteries was analyzed by qRT-PCR. Values are mean±SE. **p<0.01 vs. rapamycin group.

We detected XO in carotid arteries of different groups by western blotting. XO is activated in BI group. Rapamycin increased XO protein level in injured arteries. Co-treatment of nicorandil significantly inhibited rapamycin-induced XO protein production (p<0.01) (Figure 3A and Figure 3B). eNOS is an indicator of reendothelialization [18]. We tested eNOS mRNA expression by qRT-PCR. Rapamycin decreased eNOS production 14 days after balloon injury. Nicorandil reversed eNOS expression inhibited by rapamycin (Figure 3C).

### Nicorandil prevents CMECs apoptosis induced by rapamycin

**Figure 4:**
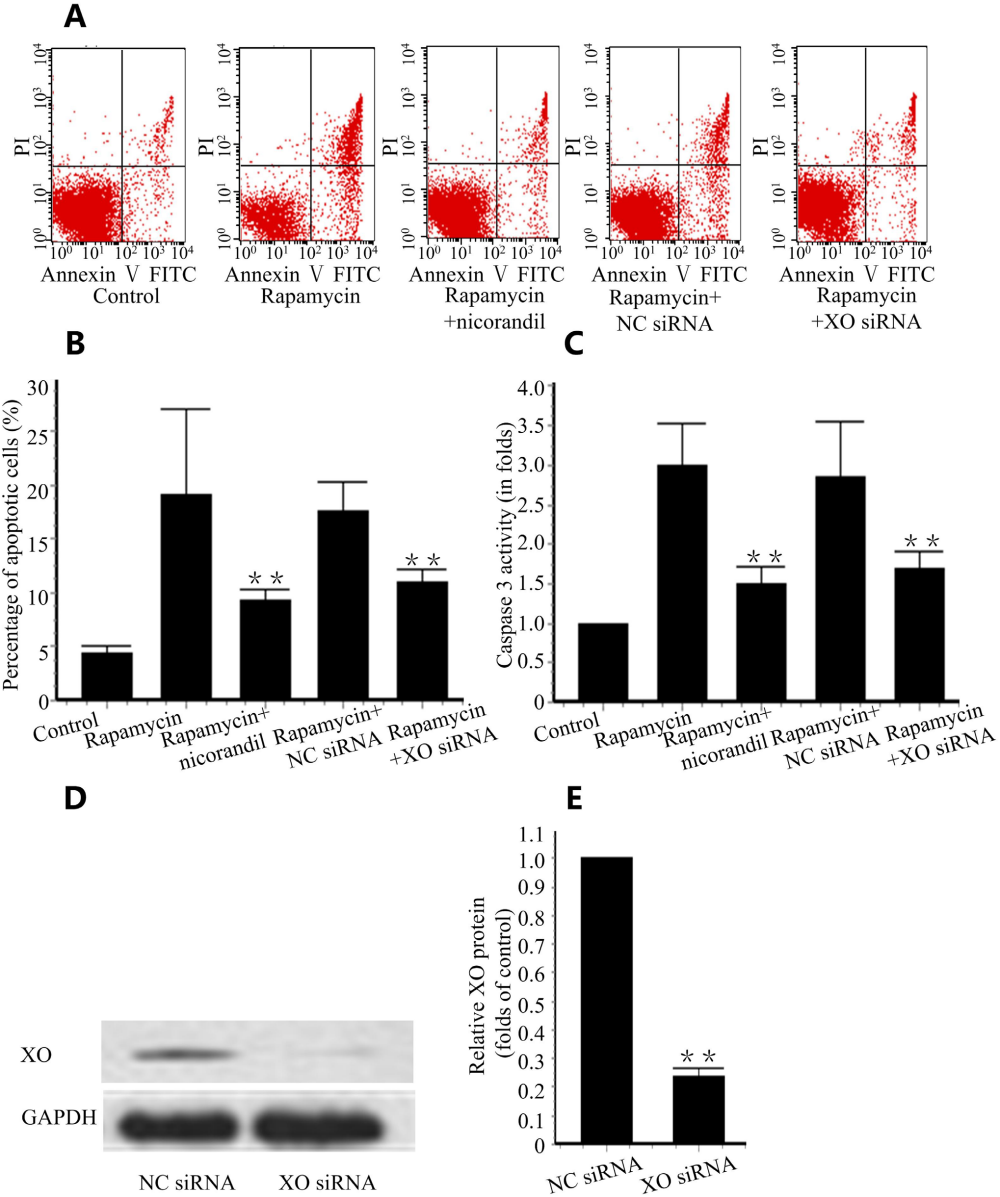
Nicorandil prevents CMECs apoptosis **A.**, CMECs were treated with rapamycin (100ng/ml) or nicorandil (100nM) for 24 h. XO is knockdown by XO siRNA. CMECs apoptosis was analyzed by Annexin V-FITC/PI staining and qualified by flow cytometry (n=3). **B.**, Summery data of percentage of apoptotic cells. Values are mean±SE. **p<0.01 vs. rapamycin group. **C.**, Summery data of caspase 3 activity in different groups. Values are mean±SE. **p<0.01 vs. rapamycin group. **D.**, XO protein was valued by western blot. CMECs were treated with negative control (NC) siRNA or XO siRNA (n=3). **E.**, Summery data of relative XO protein in CMECs. Values are mean±SE. **p<0.01 vs. NC siRNA group.

Rapamycin induced cell apoptosis in primary cultures of CMECs. Nicorandil reduced cell apoptosis induced by rapamycin from 20.04±8.59% to 9.29±0.94% (p<0.01) (Figure 4A and Figure 4B). Caspase 3 is an essential enzyme regulating cell apoptosis. Rapamycin increased caspase 3 activation in CMECs, and nicorandil inhibited this caspase 3 activation (Figure 4C). Since XO is activated in injured arteries, we tested the role of XO in apoptosis. 48h post-XO siRNA transfection, XO protein level was significantly decreased compared to cells treated with negative control siRNA (Figure 4D and Figure 4E). XO knockdown reduced cell apoptosis induced by rapamycin (p<0.01) (Figure 4A and Figure 4B). In XO-knockdown CMECs, caspase 3 activity increased by rapamycin is also reduced (p<0.01) (Figure 4C).

### Nicorandil inhibits ROS production in CMECs

**Figure 5:**
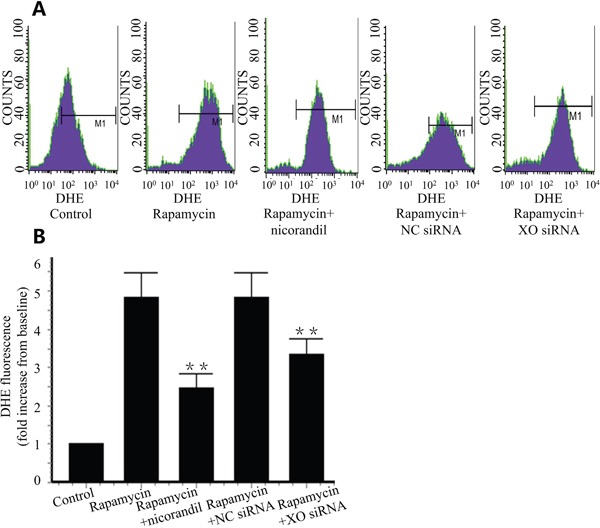
Nicorandil inhibits ROS production in CMECs **A.**, Intracellular ROS production was detected by DHE staining and assessed by flow cytometry. CMECs were incubated in rapamycin or nicorandil for 24 h (n=3). CMECs were transfected by XO siRNA or NC siRNA. **B.**, Summery data of DHE fluorescence in different groups. Values are mean±SE. **p<0.01 vs. rapamycin group.

Intracellular ROS was valued by flow cytometric analyzing of DHE staining. Rapamycin increased intracellular ROS production in CMECs by 4.85±0.64 folds compare with the control group. Nicorandil decreased the ROS level to 2.46±0.37 folds of the control group (Figure 5A and Figure 5B). To detect the role of XO in ROS production in CMECs, XO was knockdown by XO siRNA. XO knockdown significantly reduced rapamycin-induced ROS production (p<0.01) (Figure 5A and Figure 5B).

### Nicorandil promotes CMECs proliferation and migration

**Figure 6:**
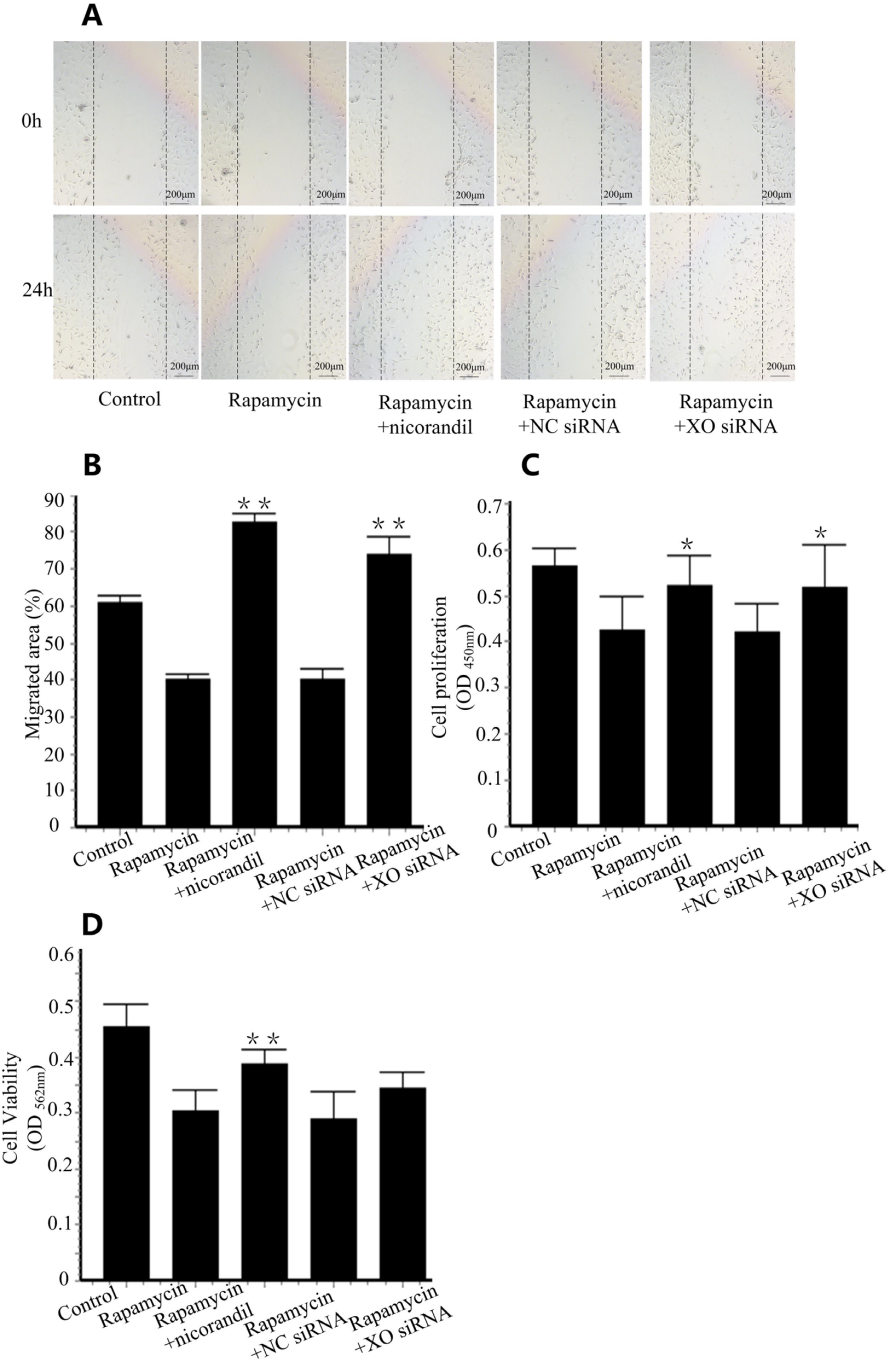
Nicorandil promotes CMECs proliferation and migration **A.**, CMECs were wounded and treated with media alone, rapamycin (100ng/ml), or rapamycin in the presence of nicorandil (100nM) for 24 h. CMECs were transfected with NC siRNA or XO siRNA (n=3). **B.**, Summary data of cell migration. Values are mean±SE. **p<0.01 vs. rapamycin group. **C.**, CMECs proliferation were determined by BrdU proliferation assay kit (n=3). Values are mean±SE. *p<0.05 vs. rapamycin group. **D.**, Cell viability was assessed by MTT assay kit (n=3). Values are mean±SE. **p<0.01 vs. rapamycin group.

Cell migration back into the denuded area was assessed after 24 h under varying conditions. Compared with control groups, rapamycin delayed CMECs migration. Co-treatment of nicorandil enhanced migration of CMECs (P<0.01) (Figure 6A and Figure 6B). 24 h rapamycin incubation also inhibited CMECs proliferation. Co-treatment of nicorandil reversed rapamycin-impaired proliferation and also increased cell viability (P<0.05) (Figure 6C and Figure 6D). When XO knockdown was achieved by XO siRNA transfection, CMECs migration impaired by rapamycin was reversed by XO siRNA (P<0.01) (Figure 6A and Figure 6B). Cell proliferation prevented by rapamycin was also enhanced by XO siRNA (P<0.05) (Figure 6C), however, cell viability reduced by rapamycin was not increased by XO siRNA (Figure 6D).

### Nicorandil activates Akt/eNOS in CMECs

**Figure 7:**
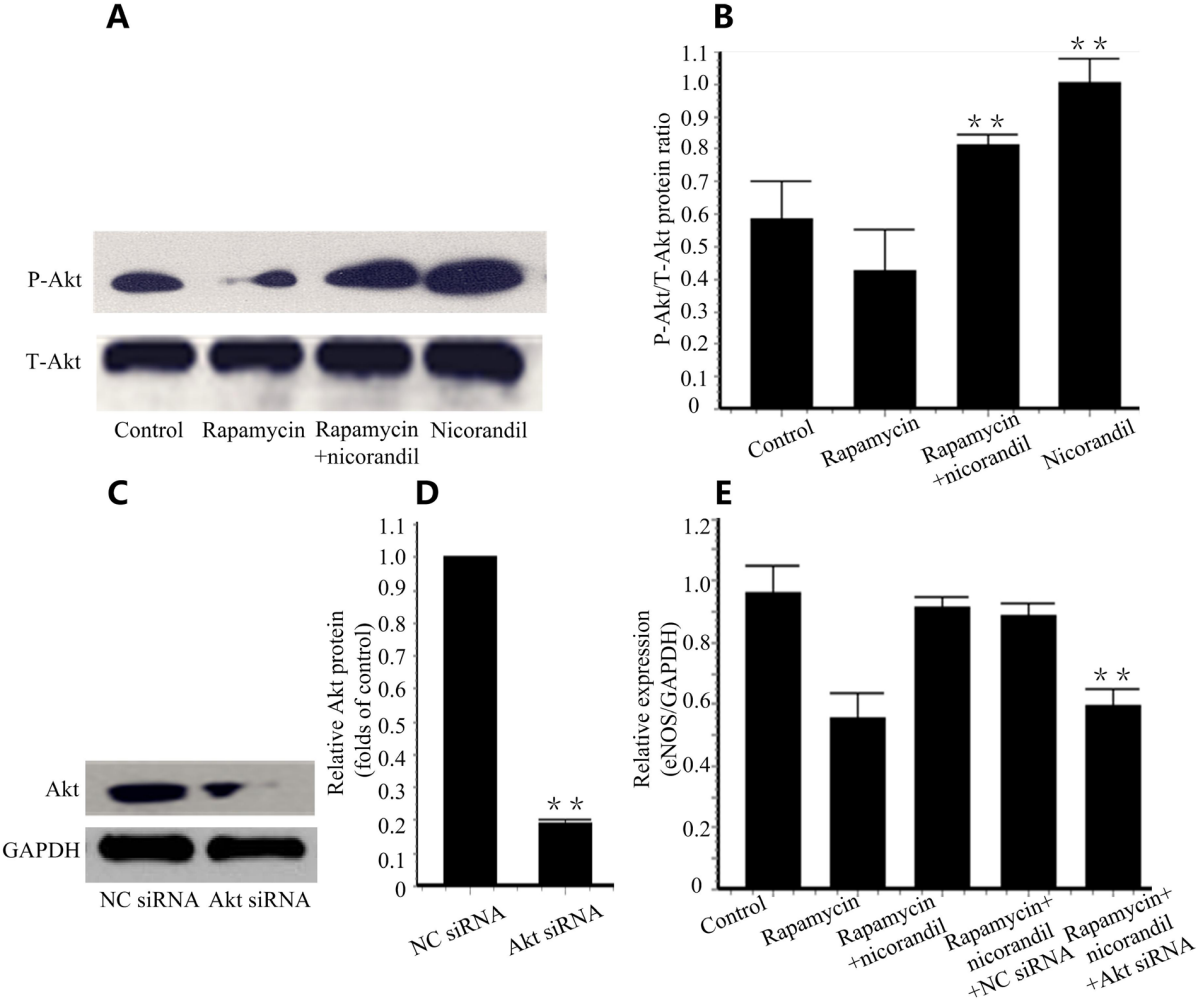
Nicorandil promotes Akt/eNOS in CMECs **A.**, Representative western blots for Akt and phosphor-Akt (Ser473) in CMECs treated with rapamycin (100ng/ml) or nicorandil (100nM) for 24h (n=3). **B.**, Quantitative analysis of Akt phosphorylation. Values are mean±SE. **p<0.01 vs. rapamycin group. **C.**, Akt was knockdown by AKt siRNA. Akt expression in CMECs were analyzed by western blot (n=3). **D.**, Summery data of relative Akt protein. Values are mean±SE. **p<0.01 vs. NC siRNA group. **E.**, eNOS mRNA expression is determined by qRT-PCR. CMECs were treated with rapamycin or nicorandil, and were transfected with NC siRNA or Akt siRNA (n=3). Values are mean±SE. **p<0.01 vs. rapamycin-nicorandil co-treatment group.

PI3K/Akt phosphorylation increases cell proliferation [19], and it is upstream of eNOS [20]. We tested Akt phosphorylation by western blotting. Expectedly, phosphorylation of Akt was inhibited by 24h of rapamycin incubation. Additional treatment of nicorandil promoted Akt phosphorylation in CMECs (P<0.01) (Figure 7A and Figure 7B). When Akt knockdown is achieved by Akt siRNA transfection (Figure 7C and Figure 7D), eNOS expression in CMECs induced by nicorandil is reduced (P<0.01) (Figure 7E).

## DISCUSSION

The principle finding is that nicorandil promotes rapamycin-impaired reendothelialization after balloon injury in rats. The main mechanism is that nicorandil decreases XO-produced ROS induced by rapamycin and nicorandil enhances eNOS expression. We also demonstrate that nicorandil enhances phosphorylation of Akt, promotes cell proliferation, increases cell migration, impedes oxidative stress and prevents apoptosis in CMECs. The beneficial effect of DESs on reducing restenosis is well established [4], primarily by preventing vascular smooth muscle cells (VSMCs) proliferation and migration. Unfortunately, DESs delay reendothelialization, and restoration of an intact endothelium is crucial for the prevention of both restenosis and stent thrombosis [1]. Rapamycin prevents eNOS expression and promotes cell apoptosis in RAECs [21], and it suppresses HUVECs proliferation and migration [22]. Protecting endothelial cells and accelerating reendothelialization after DESs implantation is of great significance for clinical application.

Rapamycin itself does not impair the intact endothelium without balloon injury (Supplementary Material Figure S1A, Figure S1B). However, rapamycin inhibited reendothelialization in balloon-injured carotid arteries, which was significantly reversed by nicorandil. Oxidative stress inhibits reendothelialization [12]. In the present study, rapamycin augmented ROS production in carotid arteries. Co-treatment of nicorandil abolished ROS elevation. ROS generation in carotid sections was affected by rapamycin or nicorandil, but not by humoral factors. In CMECs, rapamycin stimulated ROS production, which was prevented by co-treatment with nicorandil. In addition, NAC promoted reendothelialization impaired by rapamycin. Thus, nicorandil promoted reendothelialization by reducing ROS production. Multiple sources of ROS may be present in injured arteries. NADPH is activated by rapamycin in HCAECs [8]. XO is a major source of ROS in CMECs, atherosclerosis [14] and coronary disease [15]. Rapamycin directly activates XO in myeloid cells [16]. Nicorandil inhibits streptozotocin-induced XO elevation in diabetic rats [17]. We detected an increase of XO in balloon-injured carotid arteries, and rapamycin further increased XO protein level. Nicorandil reduced the XO elevation induced by balloon injury and rapamycin. Intracellular ROS production in CMECs was significantly attenuated after XO knockdown, indicating that XO is a major source of ROS induced by rapamycin. In addition, CMECs apoptosis induced by rapamycin was also greatly alleviated after XO knockdown. Nicorandil promoted reendothelialization delayed with rapamycin by suppressing XO generated ROS.

Reendothelialization depends mainly on endothelial repair of ECs adjacent to the injured area [21]. Co-treatment of nicorandil reversed CMECs proliferation and migration impaired by rapamycin. Besides, XO siRNA-transfected CMECs showed significant increase in cell proliferation and migration than those in rapamycin group. Nicorandil promoted CMECs proliferation and migration by XO inhibition. The inhibitive effect of nicorandil on ROS production could be hampered by 5-HD, a selective mitochondrial K_ATP_ antagonist [8]. Nicorandil induces endothelial cell proliferation and migration, and its effects can be blocked by 5-HD [11]. Nicorandil also blocks angiotensin II-induced XO increase by opening K_ATP_ channel in RAW264.7 cells [23]. Impairment of K_ATP_ channel triggers excessive accumulation of intracellular Ca^2+^ [24]. In our previous study, XO increase depends on elevated intracellular Ca^2+^ [13]. Mitochondrial K_ATP_ channel and intracellular Ca^2+^ may contribute to the beneficial effect of nicorandil on reendothelialization, and related experiments would be operated in our future study.

eNOS attributes to endothelium repair [25], and its mRNA expression is an indicator of reendothelialization [18]. Nicorandil is an anti-angina drug with nitrite like activity. The effects of nicorandil on eNOS are controversial. 100μM nicorandil has no significant effect on phosphorylation of eNOS in endothelial cells. Oral intake of nicorandil increases plasma NO concentration in patients with coronary slow flow [26]. 15mg/kg/day nicorandil normalizes decreased eNOS dimer/monomer ratio in diabetic rat arteries [9]. In the current study, rapamycin inhibited eNOS mRNA expression, which is consistent with previous study [27]. Co-treatment of nicorandil increased eNOS mRNA expression. Akt phosphorylation is upstream of eNOS and increases endothelial cells proliferation [20]. Rapamycin inhibits cell proliferation and Akt phosphorylation in endothelial cells [28]. Our western bolt analysis showed that phosphorylation of Akt was inhibited by rapamycin and promoted by nicorandil. When Akt was knockdown by Akt siRNA, eNOS mRNA expression increased by nicorandil was abolished. Nicorandil contributed to reendothelialization partially by activating Akt/eNOS.

Increased reendothelialization reduces intimal hyperplasia [18]. Nicorandil promotes capillary but not arterioles growth in the failing heart of hypertensive rats [29]. Nicorandil inhibits VSMCs proliferation and prevents rat pulmonary hypertension [30]. Nicorandil may inhibit intimal hyperplasia directly by inhibiting VSMCs proliferation and indirectly by promoting reendothelialization. Co-treatment of nicorandil inhibited intimal hyperplasia, but no statistical significance was observed between rapamycin and rapamycin-nicorandil co-treatment group (p>0.05). We propose that this is because rapamycin itself has sufficiently inhibited intimal hyperplasia and synergistic effects of nicorandil and rapamycin mainly rescues the deficiencies of reendothelialization. In addition, SD rats develop less intima than ApoE^−/−^ mice [31] or diabetic rats after balloon injury [32]. With more predominant intima, synergistic effects of nicorandil and rapamycin may exhibit on intimal hyperplasia.

The concentrations of nicorandil and rapamycin used in the present study were comparable with previous studies. 15mg/kg/day nicorandil was well tolerated and showed protective effects *in vivo* [9, 17]. Plasma concentration of nicorandil after a single oral intake of 30mg/kg would reach 73.7±19.9μM [8], which is comparable to the concentration of 100μM used in our *in-vitro* study. After 1 week oral intake of 15mg/kg/d nicorandil, plasma concentration would reach 0.9±0.6μM. The endothelial protective effect in the 1-week administration group was stronger than that in the single administration group, which suggests that nicorandil may accumulate in endothelial cells [8]. 100μM nicorandil is used in several related *in-vitro* studies [8, 10, 33]. Systematic concentration of rapamycin ranges from 0.4ng/ml to 2.63±0.74 ng/ml after stent deployment. Local concentration is much higher due to rapamycin's lipophilic properties and it accumulated in the vascular wall. The total arterial tissue level of rapamycin was 97.13ng/artery [2]. *In-vitro* studies use 100ng/ml or 10nM (91.4ng/ml) rapamycin to detect its effect on endothelial cells [3, 34]. Their concentrations are comparable to our rapamycin concentration.

We reveal that combining nicorandil with rapamycin results in an additional benefit-not only inhibiting intimal hyperplasia but also minimizing the deleterious effect of rapamycin on reendothelialization. INOA study indicates that nicorandil reduces major adverse cardiac events in patients with stable angina [35]. JCAD study finds that nicorandil reduces cardiovascular death in patients with ischemic heart disease [36]. Nicorandil, which potentially facilitates reendothelialization, represents a safe and effective therapeutic approach for improving the efficacy and long-term safety of patients undergoing vascular angioplasty.

## MATERIALS AND METHODS

### Rat carotid balloon injury model

All animal experiments were approved by Animal Research Committee of Chinese PLA General Hospital. Experiments were conducted in accordance with the Guide for the Care and Use of Laboratory Animals published by the US National Institutes of Health (NIH Publication No.85-23, revised 1996). Sprague-Dawley (SD) rats (male, n=70, 200-250g) were purchased from Experimental Animal Center of Chinese PLA General Hospital (approval No. SCXK 20120001). All rats were housed in a 12h light/dark cycle room at controlled temperature (23±2°C) and humidity (50-60%). Animals have a free access to regular rodent chow and water. The balloon catheter injury model was created with a 2F Fogarty catheter (Edwards Lifesciences, Irvine, CA) in the left common carotid artery [37]. Briefly, rats were anesthetized by intraperitoneal injection of pentobarbital (50mg/kg, Sigma-Aldrich, St Louis, MO, USA). The balloon was introduced through the left external carotid artery into the common carotid artery. Inflated the balloon, and then pass it through the common carotid arterial lumen 3 times. After removal of the catheter, the left external carotid artery was legated. The common carotid artery resumed blood flow. The punched area was sealed. Post operation analgesic therapy was provided by intraperitoneal administration of buprenorphine (0.05 mg/kg/day, Sigma-Aldrich, St Louis, MO, USA) for 3 days.

Rats were randomized to sham operation group, balloon injury (BI) group, rapamycin group, nicorandil group and rapamycin-nicorandil co-treatment group. Sham operation group (n=10) was conducted with an uninflated balloon and treated by gavage feeding and intraperitoneal injection with vehicle. BI group (n=10) was given BI procedure and treated by gavage feeding and intraperitoneal injection with vehicle. Rapamycin group (n=20) was given BI procedure and treated by intraperitoneal injection of rapamycin (1mg/kg/day, LC Laboratories, MA, USA) and gavage feeding with vehicle. Nicorandil group (n=10) was given BI procedure and treated by gavage feeding with nicorandil (15mg/kg/day, Chugai Pharmaceutical Co., Japan). Rapamycin-nicorandil co-treatment group (n=10) was given BI procedure and treated by intraperitoneal injection of rapamycin (1 mg/kg/day) and gavage feeding with nicorandil (15 mg/kg/day). To detect the role of ROS in reendothelialization, NAC (150 mg/kg/day, Beyotime, China) was given BI procedure and treated by gavage feeding in the rapamycin and NAC group (n=10). All groups were given different drugs for 14 days from the 1^st^ day after balloon injury.

### Morphometric analysis

To measure the reendothelialization area 14 days after BI, 5% Evans blue (Sigma-Aldrich, St Louis, MO, USA) was injected via the femoral vein. 30 min after injection, arteries were dissected out from the carotid bifurcation, and opened longitudinally to observe the Evans blue uptake macroscopically. The reendothelialization area was defined as the area not stained by Evans blue, and reendothelialization was calculated as the ratio of blue area to the total area. Images were obtained by digital camera (Cannon, Japan), and analyzed by Image J 1.49 [34]. To further evaluate the level of reendothelialization, carotid arteries were fixed in 4% paraformaldehyde, dehydrated and embedded in paraffin. 5 μm thick sections were prepared at 500 μm intervals from the carotid bifurcation. 3 serial cross sections were cut from each artery and immunohistochemical staining with PECAM-1 (CD31) (Santa Cruz, CA, USA). Images were obtained by inverted fluorescence microscope (Olympus Corporation, Tokyo, Japan). In addition, intimal hyperplasia was measured. Serial cross sections were cut from each artery and stained with hematoxylin and eosin (H&E). Images were obtained by inverted phase contrast microscope (Olympus Corporation, Tokyo, Japan). Intimal, medial and adventitial cross-sectional areas were measured by Image J 1.49.

### *In vitro* CMECs culture and drug treatment

SD rat (5-7days, 12-17g, n=60) were purchased from Experiment Animal Center of PLA General Hospital (Approval No. SCXK 20120001). CMECs were isolated from neonatal rat heart by enzyme digestion method [38]. Rats were anaesthetized by overdose of isoflurane, and the hearts were obtained. Visible connective tissue, atria, valvular tissue, right ventricular and the outer one-fourth left ventricular wall were removed. The remaining tissue were immersed in 75% ethanol for 20s, minced into pieces and digested in 0.5% collagenase type I (Gibco, Grand Island, NY) for 20min and 0.125% trypsin (Hyclone, Logan, UT) for 10min at 37°C in a shaking bath. A 100μm nylon mesh was used to filter the undigested tissue. Dissociated cells were resuspended in DMEM supplemented with 20% fetal bovine serum (FBS) (Hyclone, Logan, UT) and seeded in polystyrene flasks. CMECs were testified by IHC staining of factor VIII (Abcam, Cambridge, UK) and PECAM-1 (CD31) (Santa Cruz, CA, USA). Passage 3 to 5 cells are used for experiments.

CMECs were randomized to control group, rapamycin group, nicorandil group, and rapamycin-nicorandil co-treatment group. Rapamycin group cells were incubated in rapamycin (100ng/ml, LC Laboratories, MA, USA) for 24 h. Nicorandil group cells were incubated in nicorandil (100 μM, Sigma-Aldrich, St Louis, MO, USA) for 24 h. Rapamycin-nicorandil co-treatment group cells were incubated in rapamycin (100ng/ml) and nicorandil (100μM) for 24h.

### Measurement of ROS

ROS levels in carotid arteries were measured by dihydroethidium (DHE) staining [37]. Serial frozen fresh arteries were cut into 10 μm sections on dry ice. Sections were incubated with DHE (2 μM, Molecular Probes, Eugene, OR) at 37°C for 30min in a humidified dark chamber. Images were obtained by inverted fluorescence microscope (Olympus Corporation, Tokyo, Japan) and analyzed by Image J 1.49. Intracellular ROS measurement was conducted by DHE staining in CMECs. Cells were incubated in DHE (2 μM) for 30min, washed with PBS for 3 times, collected by trypsinization, and resuspended in PBS. Fluorescence was detected by flow cytometry (BD, Mountainview, CA) using excitation/emission wavelengths of 518/605nm.

### Cell apoptosis analysis

Cell apoptosis was analyzed by Annexin V-FITC/PI kit (Roche, Basal, Switzerland) and Caspase 3 activity assay kit (Beyotime, China). In brief, 1 × 10^6^ CMECs were collected, washed twice with PBS, and resuspended in 100 μl buffer containing 2 μl PI and 2 μl Annexin V-FITC. Then cells were kept in the dark at room temperature for 15min. Cells were analyzed by flow cytometry (BD, Mountainview, CA) [39]. Caspase 3 activity was assayed according to the manufacture's instruction. In brief, 2 × 10^6^ cells were incubated in extraction buffer for 30min on ice. Cellular extracts (30μg) were incubated with 20ng Ac-DEVD-pNA in a 96-well plate for 2 h at 37°C. Caspase 3 activity was measured spectrophotometrically (λ=405nm).

### Small interfering RNA transfection

To investigate the roles of XO and Akt in CMECs, XO or Akt is knocked down by transient transfection of small interfering RNA (siRNA). 50nM XO siRNA or Akt siRNA oligonucleotide was transfected in CMECs by using lipofectamine RNAIMAX Reagent (Invitrogen, Ireland). Rat XO siRNA sequence is 5′-CCACCUCCAAGAUUCAUAUTT-3′. Rat Akt siRNA sequence is 5′-GCAGCCAGCUCUGAUAAUATT-3′. These siRNA and negative control siRNA were purchased from GenePharma Co., Ltd. (Shanghai, China). XO or Akt knockdown is indentified by western blot. After 48 h of transfection, CMECs were harvested for other experiments.

### Cell migration and proliferation

Cell migration was investigated by wound healing assay. CEMCs (70-80% confluence) were wound with a 1.15mm diameter pipette tip. CMECs were incubated in DMEM containing 1% FBS with nicorandil (100μM) or rapamycin (100ng/ml). After 24 h, scratch photographs were taken by a phase contrast microscopy (Olympus Corporation, Tokyo, Japan). Cell proliferation was assessed by BrdU Cell Proliferation Assay Kit (Cell Signaling Technology, MA, USA) and a modified 3-(4,5-dimethyl-thiazol-2-yl)-2,5-dyphenyltertrazolium bromide assay (MTT, Sigma-Aldrich, St Louis, MO,USA) according to the supplier's instruction.

### Quantitative real-time polymerase chain reaction analysis (qRT-PCR)

Carotid arteries were frozen in liquid nitrogen. Total RNA was isolated from each 10mg carotid tissue by homogenization in 500 μl Trizol reagent (Invitrogen, Carlsbad, CA) according to the manufacturer's instructions. Total RNA from CMECs was prepared using Trizol reagent. Reverse transcription was performed using the PrimeScript reverse transcriptase (Takara Bio, Japan). cDNA was subjected to SYBR gene reagents with primers specific to the coding sequence of eNOS and GAPDH for 40 cycles. GAPDH mRNA was amplified as an internal control. This procedure was performed using the Eco^TM^ RTPCR detection system (illumine, San Diego, CA) according to the manufacturer's protocol. The primers used were as follows: 5′-GTGTGACCTGGATCCAGGCTTC-3′(sense) and 5′-TTCAGTTCAGGGATCCAGGCTG-3′ (antisense) for GAPDH; 5′-CGA TGC TCC CAA CTT GAC CA-3′(sense) and 5′-CCTCAGGATGTCCTGCACGTAG-3′(antisense) for eNOS.

### Western blot analysis

After experiments, CMECs were homogenized in RIPA lysis buffer (Beyotime, China) containing 1 × phosphatase inhibitor cocktail (Cell Signaling Technology, MA, USA). Protein (40μg) was separated by SDS-PAGE, transferred to polyvinylidene difluoride membranes, and probed with primary antibodies at 4°C overnight. Antibodies for XO (Abcam, Cambridge, UK), Akt (pan) (11E7) (Cell Signaling Technology, MA, USA), phosphor-Akt (Ser473) (D9E) (Cell Signaling Technology, MA, USA) at the concentration of 1:1000 were used. Horseradish peroxidase-conjugated secondary antibodies at the concentration of 1:5000 were incubated for 1 h at 37°C. The immunoreactive proteins on the membrane were detected by using enhanced chemiluminescence (Beyotime, China). The signal density of respective bands were qualified and normalized to GAPDH expression.

### Statistical analysis

Data were presented as mean±SE. SPSS 13.0 was used for analysis of data. One-way ANOVA with post-hoc testing were used for experiments consisting of more than two groups. If normality test failed, Kruskal-Wallis with Dunn's post-hoc test was used. Statistical comparisons were performed using the paired, two-tailed Student's t test for experiments consisting of two groups only. Results were considered statistically significant when p<0.05.

## SUPPLEMENTARY FIGURE


