# Supplementary material for: Delayed reendothelialization with rapamycin is rescued by the addition of nicorandil in balloon-injured rat carotid arteries
Source: Oncotarget. 2016 Oct 4;7(46):75926–39. doi: 10.18632/oncotarget.12444 (PMC5342788; doi:10.18632/oncotarget.12444)
Supplement: Supplementary file 1 [file oncotarget-07-75926-s001.pdf]

Delayed reendothelialization with rapamycin is rescued by the addition of nicorandil in balloon-injured rat carotid arteries

SUPPLEMENTARY FIGURE

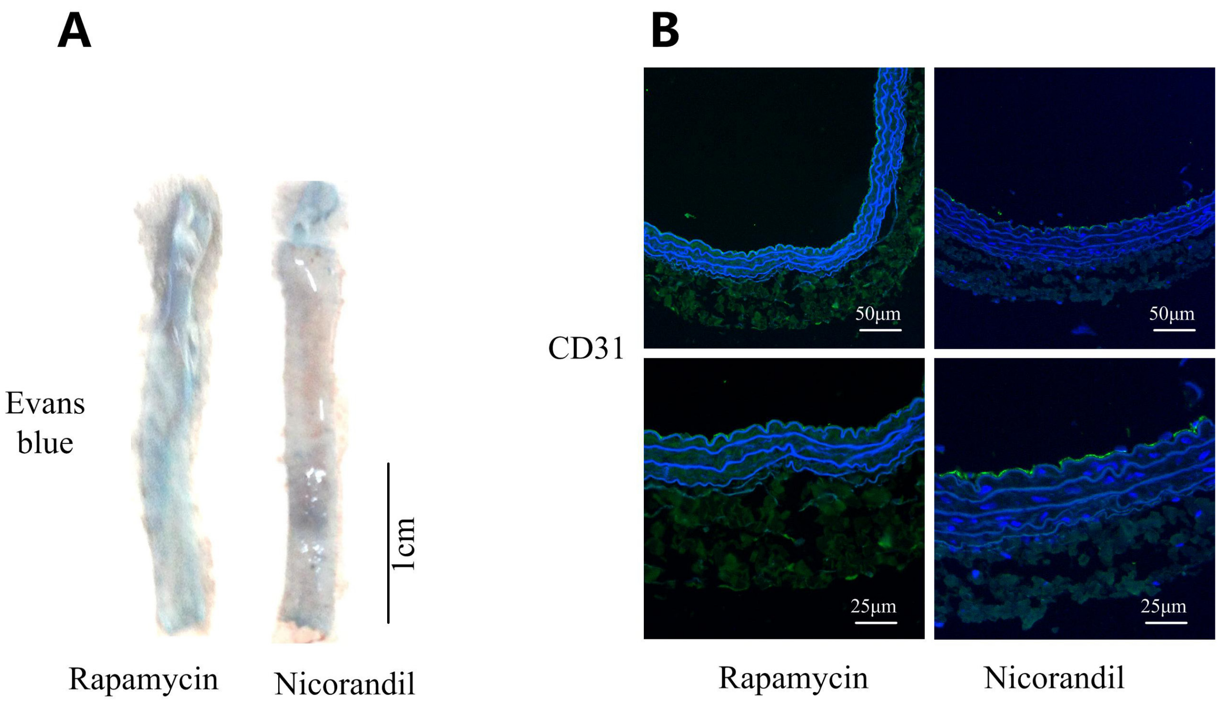

Supplementary Figure S1: Rapamycin or nicorandil does not affect the intact endothelium of carotid arteries without balloon injury (BI). A., SD rats without BI procedure were randomized to rapamycin or nicorandil. The intact endothelium area is the area that does not uptake Evans blue dye. **B.**, Immunostaining of PECAM-1 (CD31) in rats treated with rapamycin or nicorandil without BI.
